# Environmentally Friendlier Printable Conductive and Piezoresistive Sensing Materials Compatible with Conformable Electronics

**DOI:** 10.1021/acsapm.3c01151

**Published:** 2023-08-03

**Authors:** Miguel Franco, Azadeh Motealleh, Carlos M. Costa, Nikola Perinka, Clarisse Ribeiro, Carmen R Tubio, Sónia Alexandra Correia Carabineiro, Pedro Costa, Senentxu Lanceros-Méndez

**Affiliations:** † Center of Physics, University of Minho, Campus de Gualtar, 4710-057 Braga, Portugal; ‡ Institute of Science and Innovation for Bio-Sustaninability (IB-S), University of Minho, Campus de Gualtar, 4710-057 Braga, Portugal; § Abalonyx AS, Forskningsveien 1, 0373 Oslo, Norway; ∥ Institute for Polymers and Composites (IPC), University of Minho, 4800-058 Guimarães, Portugal; ⊥ BCMaterials, Basque Center for Materials, Applications and Nanostructures, UPV/EHU Science Park, 48940 Leioa, Spain; # LaPMET - Laboratory of Physics for Materials and Emergent Technologies, University of Minho, 4710-057 Braga, Portugal; ∇ LAQV-REQUIMTE, Department of Chemistry, NOVA School of Science and Technology, Universidade NOVA de Lisboa, 2829-516 Caparica, Portugal; ○ IKERBASQUE, Basque Foundation for Science, 48009 Bilbao, Spain

**Keywords:** doped graphene, conductive materials, green processing, conformable electronics, functional composites

## Abstract

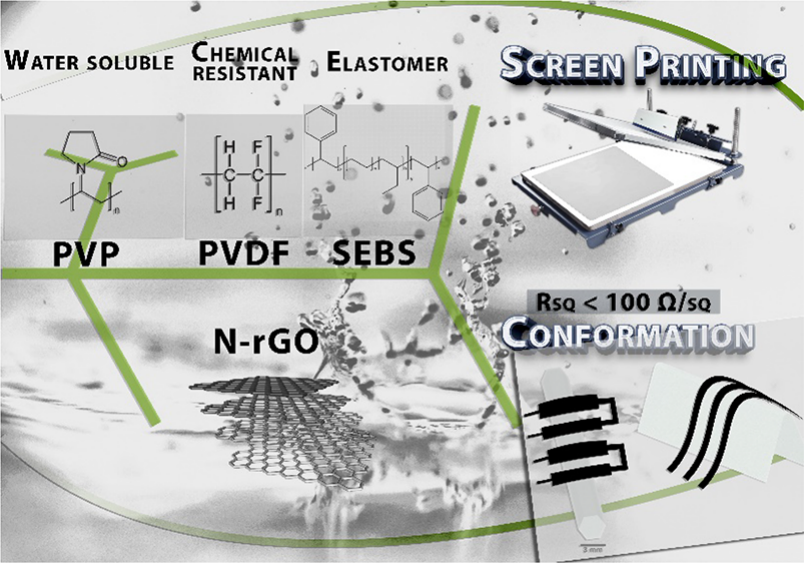

Flexible and conformable conductive composites have been developed using different polymers, including water-based polyvinylpyrrolidone (PVP), chemical-resistant polyvinylidene fluoride (PVDF), and elastomeric styrene–ethylene–butylene–styrene (SEBS) reinforced with nitrogen-doped reduced graphene oxide with suitable viscosity in composites for printable solutions with functional properties. Manufactured by screen-printing using low-toxicity solvents, leading to more environmentally friendly conductive materials, the materials present an enormous step toward functional devices. The materials were enhanced in terms of filler/binder ratio, achieving screen-printed films with a sheet resistance lower than *R*_sq_ < 100 Ω/sq. The materials are biocompatible and support bending deformations up to 10 mm with piezoresistive performance for the different polymers up to 100 bending cycles. The piezoresistive performance of the SEBS binder is greater than double that the other composites, with a gauge factor near 4. Thermoforming was applied to all materials, with the PVP-based ones showing the lowest electrical resistance after the bending process. These conductive materials open a path for developing sustainable and functional devices for printable and conformable electronics.

## 1 Introduction

Over the last few years, innovative solutions in electronic device technologies have been demanded.^1,2^ In particular, paradigms related to digitalization, the Internet of Things (IoT) and Industry 4.0 revolutionizes the requirements for sensing and functional materials in terms of increased performance, reduced environmental impact, and simplified processing and integration.^3^ Additive manufacturing technologies are thus increasingly being established to develop smart and functional materials with tailored properties for electronic applications, leading to new generations of lightweight multifunctionality materials with improved integration and functionality.^3−5^ Among the different additive manufacturing technologies, screen- and inkjet printing are the most common techniques used for printed electronics.^6^ Screen-printing is the most commonly used technique for printed electronics, with low complexity, scalability, and high throughput, presenting a huge potential for mass production of large-area electronics at a low cost while allowing complex patterns^4,7,8^ in a wide range of polymeric substrates.^9−11^ Further, printing technologies can be combined with additional processing strategies to further tailor electronic devices with innovative geometries. In particular, the thermoforming process allows for the development of curved-shape electronics with high-precision geometry and fast processing.^12^ Thus, the combination of additive manufacturing techniques with the thermoforming process will allow advanced functional electronic solutions with tailor-made designs and improved integration.^13^

Nevertheless, for this technique to be sustainable on a large scale, processing and the materials used for composite development must rely on materials that present low toxicity for human health and the environment. In particular, it is urgent to avoid the use of toxic solvents and to explore greener alternatives^14^ to the ones commonly used nowadays for a large range of polymers. Further, those alternative solvents should show low boiling temperature, high vapor pressure, and low surface tension to lead to stable materials with good processability, which are key issues for the scalability of the processes.^15^ Low-toxicity and/or bio-based solvents, such as alcohol, *p*-cymene or cyrene, cyclopentyl methyl ether (CPME), 2-methyl tetrahydrofuran (2-MeTHF), or dimethyl sulfoxide (DMSO), are increasingly used to develop printable polymer composites for high-performance devices.^16,17^

One of the most required functionalities for multifunctional composites is electrical conductivity, which can be achieved using an electrically conductive filler within a polymer matrix. Composites with filler content above the percolation threshold have piezoresistive properties that can be tailored for sensor applications from low to larger strains.^18−21^ Graphene is a two-dimensional carbon lattice, with good electrical, mechanical, and thermal properties,^22,23^ presenting a larger surface area,^24^ which has become a suitable material for the development of flexible conductive patterns.^25−28^ Unlike metals, which may be scarce and lead to environmental issues during extraction and refining, graphene can be produced from graphite, which is an abundant material with a scalable and sustainable production capability, and some studies show that composites reinforced with graphene are cytocompatible for different material variations and contents.^29^ The electrical properties of graphene can be tailored with chemical or physical treatments.^30^ Among the different graphene variations, nitrogen-doped reduced graphene oxide (N-rGO) shows superior electrical conductivity and good compatibility with polymer matrices.^29^

In the present work, different conductive graphene-based printable materials were prepared using polyvinylpyrrolidone (PVP), polyvinylidene fluoride (PVDF), or styrene–ethylene–butylene–styrene (SEBS) and environmentally friendly solvents. The materials were optimized to obtain conductive printed films, tailoring the pattern and line thickness, and their printing characteristics and mechanical properties after the thermoforming were also performed as structural sensing materials, evaluating their piezoresistive performance in bending mode.

In this way, three different polymers have been used, with different overall characteristics in order to tailor materials for specific application areas. The electroactive PVDF is a hydrophobic thermoplastic fluoropolymer with high chemical, mechanical, thermal, and UV radiation resistance,^31,32^ being interesting for sensor applications. PVDF is commonly processed from solution in dimethylformamide (DMF); however, due to its toxicity, solvents with lower noxiousness must be used, making dimethyl propylene urea (DMPU) a greener alternative.^33^ For large strain sensor applications, the SEBS elastomer, which presents large elasticity, excellent heat, and UV resistance, has been used.^34^ Toluene is commonly used to dissolve SEBS, but being a toxic solvent, *p*-cymene has been used as an alternative. *p*-Cymene is an alkyl-substituted aromatic compound naturally occurring in essential oils^35^ that can be obtained in large amounts as a side product of the cellulose and citrus industry. PVP is a water-soluble thermoplastic polymer that is also inert, nontoxic, temperature-resistant, pH-stable, biocompatible, and biodegradable,^36^ allowing water-based ink formulations.

## 2 Results and Discussion

### 2.1 Morphological and Chemical Characterization

**Figure 1:**
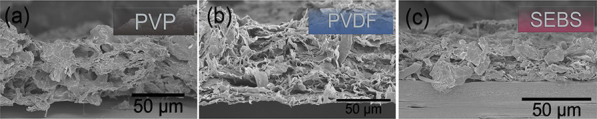
Cross-sectional SEM photographs of the 5-layer screen-printed films with a filler binder ratio of 1:2 for: (a) PVP, (b) PVDF, and (c) SEBS.

The morphology of the N-rGO screen-printed films with different polymer binders and filler content was evaluated by SEM images in cross-sectional mode, as presented in Figure 1.

**Figure 2:**
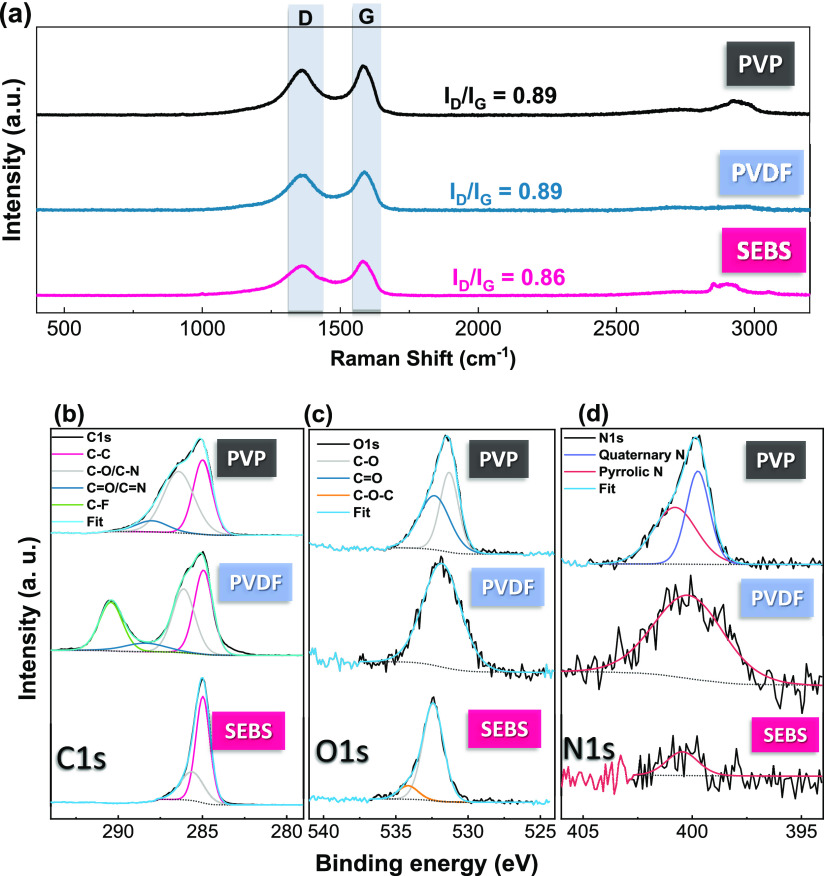
Printed 5-layer films prepare with a filler:binder ratio of 1:2: (a) Raman spectra for each composite. XPS spectra and respective peak deconvolution for the different materials: (b) C 1s; (c) O 1s; and (d) N 1s.

As observed in Figure 1, no significant differences were found between the different samples. The screen-printed films present a noncompact structure with pores of a few μm in size for PVDF and PVP, decreasing for the SEBS composite. PVDF and PVP show thicker thickness for the 5-layer films (50–60 μm), whereas the SEBS material, with the same number of layers, has a thinner thickness of 30–35 μm, due to the lower porosity and pore size (Figure 1). Compact graphene layer films can decrease their intrinsic sheet resistance, unlike porous structures.^10^ Chemical characterization of the films was performed by Raman and XPS analysis, both presented in Figure 2, for printed films with five layers and a filler:binder ratio of 1:2.

Raman spectroscopy was used to analyze the characteristics D and G bands of carbon materials with varying polymer binding and processing conditions (Figure 2a). The G band, associated with crystalline graphene, is located at 1580 cm^–1^, whereas the D band, is associated with defects such as honeycomb structure about the sheet edges, vacancies, and amorphous carbon is located at 1353 cm^–1^.^19^ No significant variations of the ratio between the D and G bands were found among the different samples, with a ratio lower than 1 and a variation below 13% for the different samples, indicating that the processing conditions do not affect the quality of the material. The ratio between the D and G bands is slightly lower in comparison to previous work,^29^ due to the higher reduction temperature of the used N-rGO (1100 °C, compared to 900 °C in the previous work^29^). The nitrogen-doped rGO has a D/G ratio between 0.86 and 0.89. The addition of nitrogen increases the defect band when compared to rGO materials.^37,38^

XPS allows the evaluation of the chemical composition of the printed films, both quantitatively and qualitatively (Figure 2b–d). The obtained spectra for the carbon region can be deconvoluted into different peaks, each one correlated with specific chemical bonds. The bands observed are C=C and C–C (sp^2^, 284.5 eV), C–O (ether and hydroxyl, 286.0 eV), C–N (286.0 eV), C=O (288.0 eV), and, in the case of PVDF, C–F_2_ (292.0 eV).^19,39,40^

Analyzing the O 1s peak at the 530 eV region (Figure 2c), the C–O and C=O bonds are identified between 531.0 and 532.0 or 532.0 and 533.0 eV, respectively.^19^ Also, a broader peak, found between 533.5 and 534.0 eV, can be linked to C–O–C (lactone groups).^41^ PVP and PVDF samples annealed at 100 °C do not have that peak in their O 1s spectra.

Despite the reduced graphene oxide being doped with nitrogen, the addition of polymer and the existence of oxygen species in the samples mask the C–N and C=N bonds. Figure 2d shows the N 1s spectra. For PVP, it shows two main peaks: a smaller one at 400 eV, corresponding to quaternary N, and a larger one at 401 eV, corresponding to pyrrolic N. For PVDF and SEBS, only pyrrolic N was found.^42^

### 2.2 Printability of the Different Ink Compositions

**Figure 3:**
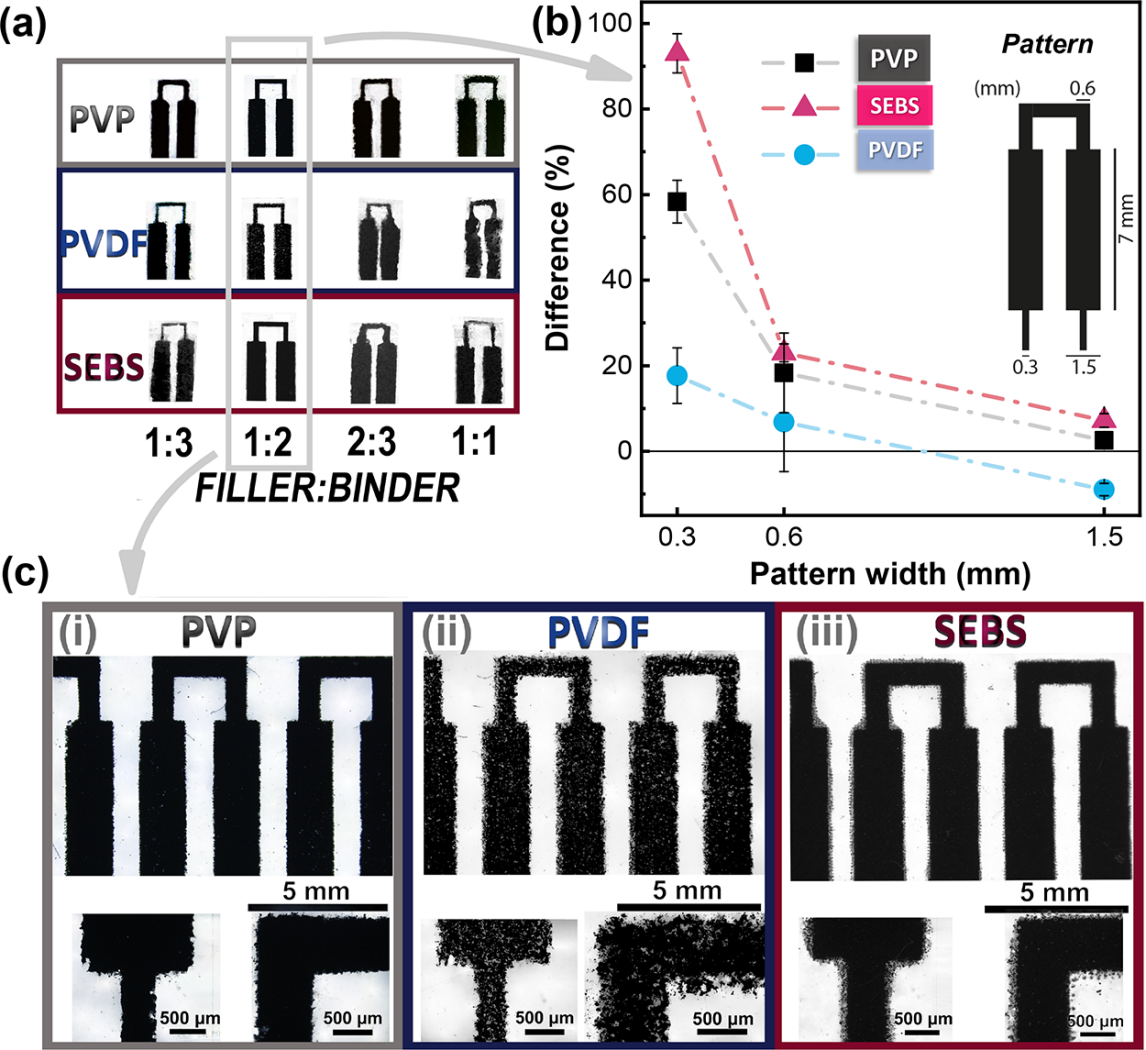
(a) Photographs of the screen-printed patters using inks with varying filler:binder ratio. For the ink with a 1:2 filler:binder ratio: (b) difference between the obtained printed pad dimensions and the one of the screen and (c) patterns with a magnification of 8× and 35× for (i) PVP, (ii) PVDF, and (iii) SEBS.

The overall properties of the printed materials depend on the polymer binder, filler, and solvent used, as well as their ratios (filler:polymer ratio and solute:solvent ratio), which influence the ink’s resolution on printed patterns, where low solute content shows low resolution and higher contents show higher rugosity and low homogeneous samples with voids or holes.^29,43^ Therefore, the influence of different polymer/solvent formulations was studied. A pad pattern was designed (Figure 3) with lines with widths of 300, 600, and 1500 μm in order to evaluate the printing quality of the materials.Figure 3 shows the printability of the PVP, PVDF, and SEBS based composites, with different magnifications (8× and 35×) obtained after 5 printing steps and varying the binder:filler ratio for the different formulations. It is proven that the developed inks, formulated with environmentally friendly solvents and different polymeric binders, can be applied by screen-printing to obtain patterns with different geometries and lines as thin as 300 μm (Figure 3a).

Figure 3a shows the printed patterns for all polymer binders when varying the filler:binder ratio. The 1:2 ratio shows the best printing quality for all the polymers. Figure 3b,c shows the printing resolution and optical microscopy photographs, respectively, for the filler binder ratio of 1:2. Among the different binders, SEBS and PVP show excellent coverage and well-defined patterns for line thicknesses of 600 and 1500 μm. As a water formulation, PVP shows remarkable printing definition capability. No intervals, voids or holes are found in the lines, and the width is uniform. SEBS based inks allow patterns down to 300 μm with great uniformity, although with a higher width discrepancy (close to 100% at 300 μm).

PVDF based inks generate patterns with similar printable definition as SEBS (Figure 3b) but with worse coverage with respect to PVP or SEBS formulations (Figure 3c). In fact, the difficulty of formulating a good PVDF based ink for printing techniques is well known from the literature.^44^ With these results, the water-based PVP formulation shows the best printing quality with good coverage and a resolution of 300 μm.

### 2.3 Adhesion Properties

**Figure 4:**
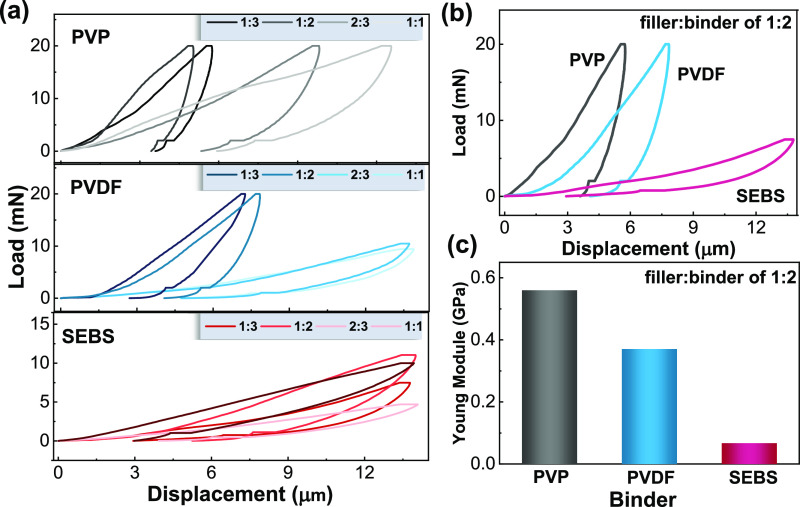
Applied load as a function of the displacement during the indentation process for (a) PVDF, PVP, and SEBS based films with different filler:binder ratios; (b) PVDF, PVP, and SEBS with a filler:binder ratio of 1:2; and (c) Young’s modulus, calculated from the indentation curves, for the films prepared with different binders for a filler:binder formulation of 1:2.

The nature of the polymer binder can greatly change the adhesion and mechanical properties of the printed materials. Further, the inclusion of fillers also changes the adhesion and mechanical properties with respect to the pristine materials.^29^ Adhesion is a key parameter to evaluate the suitability for applications of the films printed on substrates, especially for thermoforming into structural devices. Therefore, adhesion of the screen-printed films to the commercial Kapton substrate was evaluated by microindentation (Figure 4). This technique generates a load–displacement curve, from which the displacement depth and Young’s modulus values can be obtained.^45^

As shown in Figure 4a, the effect of increasing filler content in the material leads to an increase in displacement when a maximum force is applied, for the different binders. With respect to PVP, the displacement varies from 5.0 to 13 μm for 1:3 and 1:1 filler:binder ratios, respectively. The large displacement between 1:2 and 2:3 filler:binder ratios means that, for ratios higher than 1:2, the repulsive forces of the N-rGO sheets cannot be effectively balanced by the polymer binder, diminishing the adhesion properties.^46^ The same trend occurs for the samples with PVDF and SEBS as binders, with the elastomeric SEBS having a lower load and higher displacement compared with thermoplastic binders. Therefore, for the N-rGO filler, a filler:binder ratio of 1:2 is the highest value, allowing good adhesion properties.

Figure 4b shows the load–displacement curves for the different polymer binders with the same filler:binder ratio of 1:2. As expected, depending on the mechanical properties of the binder, distinct mechanical characteristics are observed. The thermoplastic PVP based films show the lowest displacement of 5.5 μm, followed by PVDF films, with a displacement of 7.8 μm (42% larger than PVP). The elastomeric SEBS films show a displacement of 13 μm (136% larger than the PVP). Both PVP and PVDF show maximum applied forces of 20 mN (limited by software, in order not to have the influence of the substrate). For SEBS, 14 mN was enough to displace 13 μm. The observed differences are related to the different nature of the polymer binders, with the SEBS elastomer being softer, compared to the PVP and PVDF thermoplastics.

The Young’s modulus can be quantitatively calculated from the displacement curves, and the obtained values are shown in Figure 4c. For the different binders, the Young’s modulus presents values in the same order of magnitude for PVDF and PVP, 370 and 570 MPa, respectively, decreasing to 67 MPa for the softer SEBS binder. The polymer binder, filler:binder ratio, and solvent used in the ink formulation influence the mechanical properties of the printed materials.^29^

Overall, thermoplastic PVP based materials show better adhesion properties, having the lowest displacement values among the tested polymer binders.

### 2.4 Electrical Properties of the Printed Patterns

**Figure 5:**
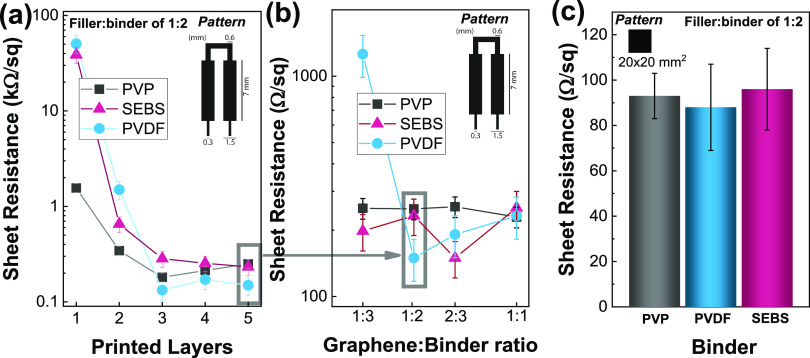
Sheet resistance of the screen-printed pads for PVP-, SEBS-, and PVDF-based films: (a) for pads as a function of the number of printed layers; (b) for pads as a function of the filler:binder ratio for 5 printing steps; and (c) for a 20 mm square obtained after five printing steps using a filler:binder ratio of 1:2.

In order to evaluate the suitability as conductive materials obtained by screen-printing, the electrical sheet resistance of the films was evaluated by the 4-point method (eq 1) on 1.5 × 7 mm^2^ pads. The effect of the number of printed layers on the electrical conductivity was evaluated for a filler:binder ratio of 1:2, as shown in Figure 5. Figure 5a presents the sheet resistance for films obtained with 1 to 5 printing layers, with the resistance decreasing with increasing the number of printed layers and the corresponding thickness of the samples. The printed materials with one layer show sheet resistances near 40 kΩ/sq for PVDF and SEBS and 1.5 kΩ/sq for PVP, decreasing below 300 Ω/sq, independently of the polymer binder, for printed samples with three layers, which show low sheet resistance, even presenting a further slight decrease up to five layers. The minimum resistance obtained is similar for all materials near 100 Ω/sq for materials using PVDF as binder.

Figure 5b shows that, apart from PVDF when using a filler:binder ratio of 1:3, there are no significant variations in the sheet resistance of the films for samples obtained after five printed layers. Hence, the sheet resistance is mainly determined by the filler itself when above the percolation threshold. As the filler:binder ratio and the printed steps are fixed, 1:2 and 5, respectively, and using a larger square to ensure the geometry has minimal interference on the sheet resistance value, the variation of the resistance among the samples obtained with the different binders is lower than 10% (Figure 5c). Nonetheless, PVDF shows the lowest sheet resistance among the three (88 ± 19 Ω/sq), followed by PVP (93 ± 10 Ω/sq) and SEBS (96 ± 18 Ω/sq). The pattern area also has a slight influence on the electrical properties, increasing for smaller areas in screen-printing technology.

It has been reported previously that printed nitrogen-doped graphene composites with PVP as binder lead to a sheet resistance of 3.9 kΩ/sq.^29^ Comparing with related formulations, a water-based formulation using cellulose (CMC) as binder achieved a sheet resistance of 197 Ω/sq when using 14% of rGO and a filler binder ratio of 9:1,^47^ still 2× higher, when compared to the PVP water-based formulation in this work. Thus, this work presents a high-conductive ink that was developed and processed by printing technologies.

The used process, binder content, and printing process, combined with the intrinsic properties of doped graphene, limit the resistivity of the materials. Taking these results in consideration, the filler:binder ratio that provides a suitable combination of adhesion properties, printing quality, and electrical conductivity is the filler:binder ratio of 1:2, regardless of the polymer used in this work. Therefore, the 1:2 ratio was chosen for the remaining experiments.

### 2.5 Piezoresistive Sensing Performance

**Figure 6:**
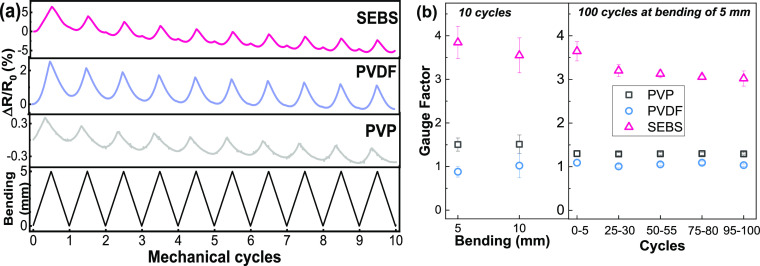
(a) Electrical resistance variation under applied maximum bending of 5 mm over 10 cycles for all samples. (b) Piezoresistive GF for 5 and 10 mm of bending for 10 cycles and as a function of the number of bending cycles for 100 cycles at a maximum deformation of 5 mm.

The developed materials present great overall properties for printed electronic applications: low resistivity, good adhesion, flexibility (for all binders), and stretchability (for SEBS binder). The multifunctionality of the printed materials for the different binders was demonstrated for piezoresistive sensing applications. Piezoresistive tests were carried out in material samples with filler:binder ratios of 1:2 and 7 × 1.5 mm^2^ patterns, under 5 and 10 mm maximum mechanical bending (along the vertical direction) with simultaneous measuring of the electrical resistance variation (Figure 6a). The variation of the electrical resistance follows the applied stimulus for the different samples and is stable for over 100 cycles, as shown in Figure 6b. The relative resistance variation under bending is larger for the soft binder and decreasing for the rigid ones (Figure 6a), as determined by the gauge factor (GF) in Figure 6b. In all cases, the electrical resistance varies linearly with the applied bending deformation.

The samples with SEBS binder show a GF = 3.8, decreasing to 0.8 < GF < 1.5 for samples with PVDF and PVP. The mechanical properties of the binder materials critically influence the sensitivity of the printed films for sensing applications. The piezoresistive response of the more rigid thermoplastic films is dominated by the geometrical factor (1 + 2υ),^19^ which is near 1.6–1.7 for PVP and PVDF, respectively. The geometric factor for SEBS is about 1.80, revealing that the intrinsic resistance variations in soft material increase its sensitivity (nearly 4 times higher) compared to the thermoplastic binder,^48^ presenting also a good linearity with applied stimulus during the piezoresistive tests. Although SEBS-based materials show greater sensitivity, PVDF shows the most stable electrical behavior under repeated cycling. Thus, the piezoresistive response of the printed materials for 100 cycles at a maximum bending of 5 mm shows a slight decrease in GF for the SEBS samples, being stable for the thermoplastic ones. Overall, the developed printed conductive materials tolerate mechanical bending with no significant electrical resistance variation degradation, being able to self-sensing evaluate that bending through the piezoresistive response.

### 2.6 Thermoforming of the Functional Printed Materials into Structural Parts

Recognizing the excellent electrical, mechanical, and functional properties of the materials, one suitable application is thermoforming, allowing to translate the 2D patterns into 3D structural and functional devices. Using commercial polyethylene terephthalate glycol (PETG) with a thickness of 0.5 mm as substrate with a conformation temperature of 150 °C allows the thermoforming of the printed patters without their thermal degradation.

**Figure 7:**
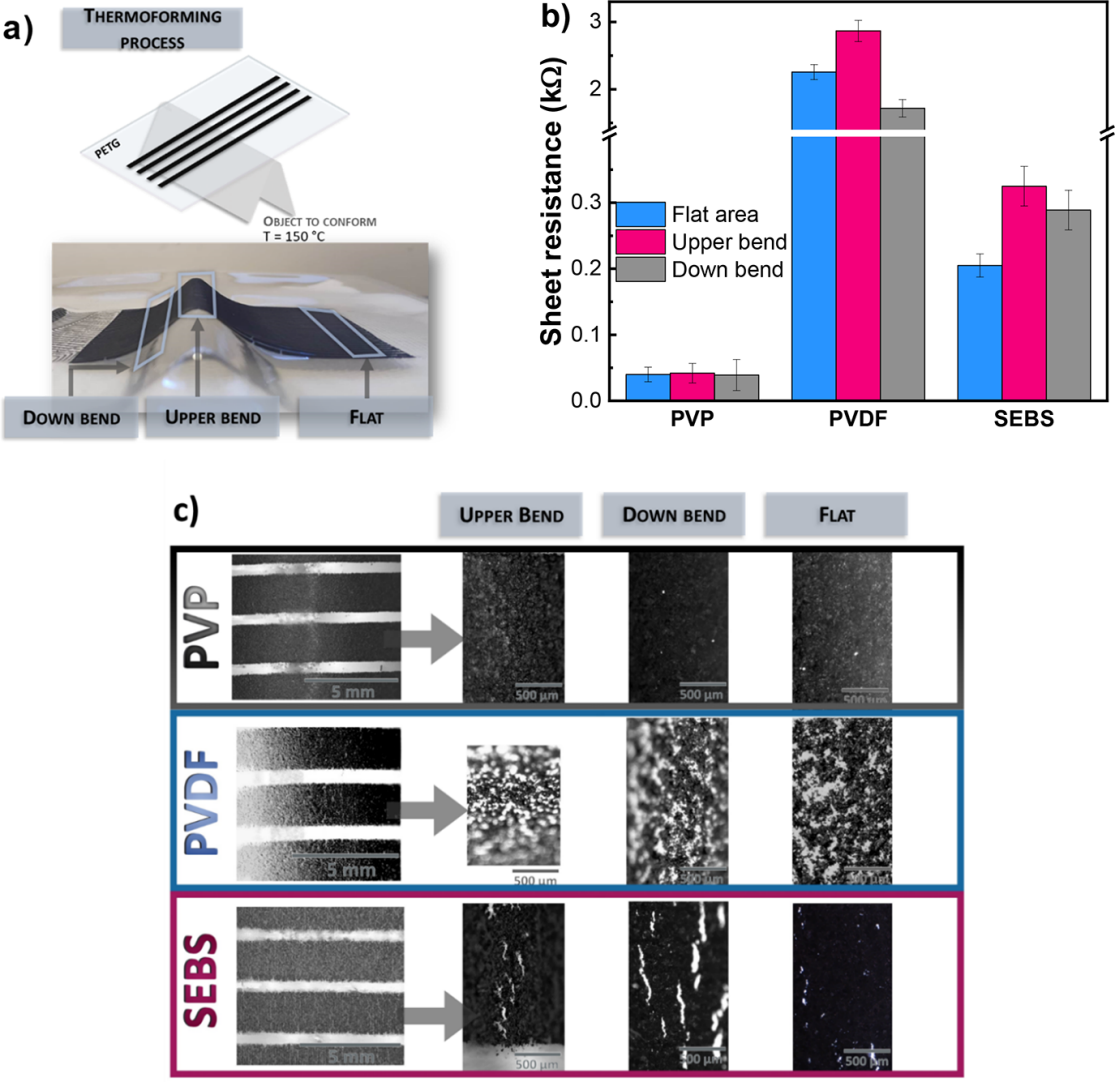
(a) Scheme of the screen-printed pattern on PETG substrate after thermoforming at 150 °C. (b) Sheet resistance values (average of the measures in five lines) measured on different sections. (c) Optical microscope photographs of the conformed films with a magnification of 8×. The lines were obtained with a filler:binder ratio of 1:2 and after five printing steps.

To evaluate the performance of the functionality of the screen-printed materials after the thermoforming process, a 40 line pattern with 2 × 95 mm of width × length was printed over the PETG substrate by screen-printing using five printing steps (Figure 7a). The printed materials are the ones with a filler:binder ratio of 1:2 for the three different polymer binders and were thermoformed using a triangular shape support with 35 mm of base per 15 mm of height (Figure 7a). Despite the thermoforming into an object with high relief, all materials continue to be fully functional, as demonstrated by the sheet resistance of the materials (Figure 7b).

The electrical resistance of the materials printed over PETG after thermoforming is shown in Figure 7b, measured in lines with 2 mm of width and a length of 8 to 11 mm with silver paint electrodes at three different zones of the 3D structure: flat area, upper bend, and down bend (Figure 7a). The sheet resistance of the materials depends not only on their intrinsic properties but also on the applied bending during thermoforming (Figure 7b). Printed PVP shows the lowest sheet resistance values with near 40 Ω/sq in the flat area, with similar values for the bent parts (top and bottom), thus demonstrating that it is not to be affected by the temperature bending process. The thermoplastic PVDF presents higher sheet resistance near 2.2 kΩ/sq at the flat area, increasing the resistance in top and down bent parts, presenting the top zone the highest value. Elastomeric materials with SEBS as binder present higher sheet resistance than PVP material, with a sheet resistance of 200 Ω/sq at the flat area, increasing for both bent zones, with 325 Ω/sq for the top bend part.

Figure 7c summarizes the morphology of the films after thermoforming. The PVP sample shows no cracks and a uniform film with good printing definition for both flat and bent zones. On the other hand, SEBS films show multiple small cracks across the printed line for distinct evaluated zones, leading to an increase in resistance. Multiple cracks and voids can also be found in PVDF materials. Besides the mechanical properties of the PVDF, the interaction between the substrate and the film (also the solvent) is poorer than for the other binders.

Thus, PVP presents excellent adhesion, electrical, and mechanical properties for the thermoforming of environmentally friendly screen-printed materials, with excellent conductive and piezoresistive responses.

### 2.7 Cytotoxicity of the Printed Patterns

**Figure 8:**
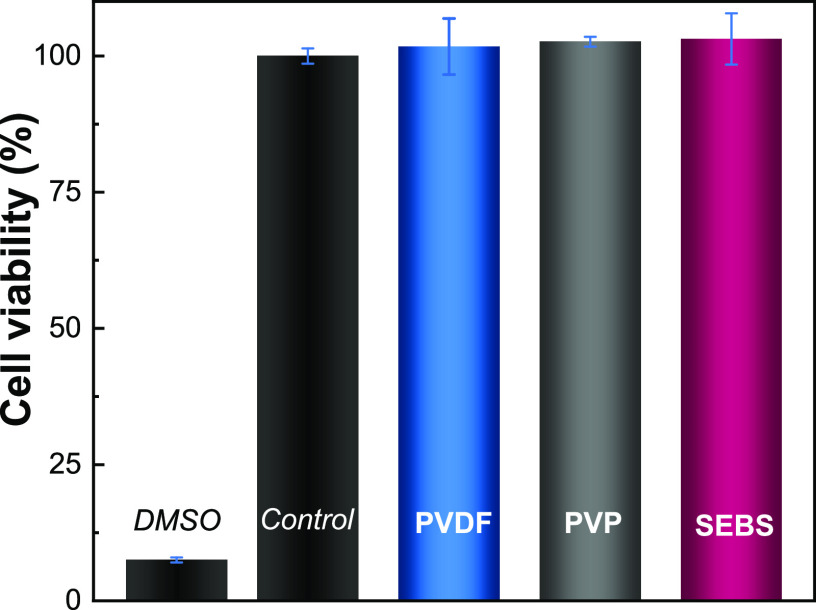
Cytotoxicity results of the L929 cells in contact with the as-prepared extraction media exposed to the different screen-printed materials prepared with a filler:binder ratio of 1:2 for 24 h, with the corresponding controls.

Conductive materials processed using environmentally friendly approaches can be explored in biomedical applications. Hence, *in vitro* cytotoxicity tests were performed, and the results are presented in Figure 8 for different polymer binders.

According to ISO standard 10993-5, samples are considered cytotoxic when cells suffer a viability reduction larger than 30%. The toxicity of N-rGO is dependent on dose and size.^49^ In the present case, the obtained cell viability values are all higher than 70%, confirming the cytocompatibility of the samples independently of the processed conditions and composition. Thus, the inclusion of the fillers and the processing with the selected polymers do not modify the biocompatibility and absence of toxicity of the pristine polymers PVP,^36^ PVDF,^50^ and SEBS,^51^ which agree with the results obtained in our work. This confirms the viability of using the developed materials for biomedical applications.

## 3 Conclusions

Flexible, conductive polymer-based materials reinforced with nitrogen-doped graphene have been optimized for screen-printing. To cover a wide range of applications, UV-resistant, water-soluble, and stretchable polymers, thermoplastics PVDF and PVP, and elastomeric SEBS were used as polymer binders for the developed inks. Together with the conductive properties, the developed materials present a piezoresistive sensing response and are capable of withstanding the thermoforming process, transforming 2D materials into functional 3D structural devices. Environmental-friendly solvents were employed to develop the ink formulations. Printability, adhesion, mechanical properties, low electrical resistance, and piezoresistive properties were achieved in all developed composites.

The patterns can be printed by all materials with similar electrical properties after five step layers, with the sheet resistance lower than *R*_sq_ < 100 Ω/sq. These materials can also be used as functional materials for bending mechanical sensors with a GF ≈ 1 from 1.5 for the thermoplastic polymers and GF ≈ 3 to 4 for the elastomeric SEBS. Finally, the materials were printed over commercial PETG substrates to create structural components through the thermoforming technique. PVP reinforced with N-rGO is the most appropriate material for the thermoforming process, with improved adhesion and lower resistance.

## 4 Experimental Section

### 4.1 Materials

PVP (average *M*_w_ ≈ 1.3 × 10^6^ g/mol, Sigma Aldrich, reference 437190), SEBS (Calprene H6120, 68/32 ethylene–butylene/styrene ratio) and PVDF (Solef 6010, density of 1.8 g/cm^3^), have been used as polymer binders. Nitrogen-doped reduced graphene oxide (N-rGO) from Abalonyx (1 nm thickness per layer and a flake size of 5 μm) was used as conductive filler. The N-rGO was annealed at 1100 °C during the synthesis process. The electrical conductivity of N-rGO is 3.3 S cm^–1^, and the surface area (m^2^ g^–1^) and apparent density (g cm^–1^) are 200–250 and 46–52, respectively, as provided by the supplier. Three solvents were used: ultrapure water (Mili-Q integral 5, with a resistivity of 15 MΩ.cm) for PVP, *p*-cymene (98%, Sigma Aldrich, reference C121452) for SEBS and DMPU (min. 99.0% purity, BASF) for PVDF.

### 4.2 Preparation of Environmentally Friendly Conductive Graphene-Based Inks and Printed Films

**Figure 9:**
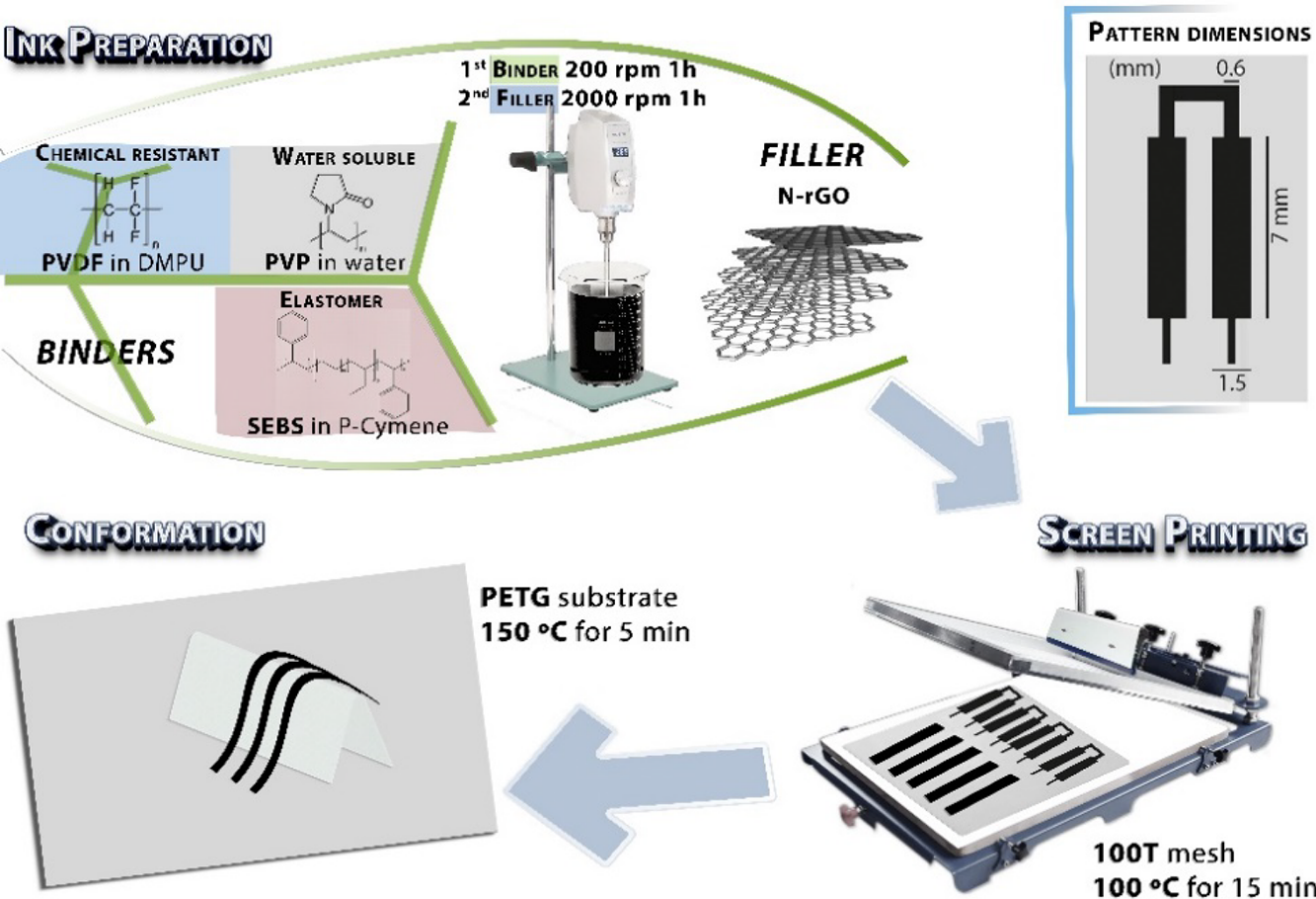
Experimental procedure for N-rGO based inks and films preparation using PVDF, PVP, and SEBS as polymer binders. The films were screen-printed in 20 × 20 mm squares and (1.5 × 70 mm) pads.

The preparation of the inks and films follows the general guidelines presented in detail in ref (29): it first starts with the complete dissolution of the polymer binders in the corresponding solvent (PVP in ultrapure water, SEBS in P-cymene, and PVDF in DMPU) under mechanical mixing (Heldolph D-91126) at 200 rpm for about 1 h, at room temperature. Next, the corresponding amount of N-rGO (Table 1) was continuously added in small portions to the solvent/polymer solution. Then, the material was kept under further mechanical stirring for 1 h at 2000 rpm for proper graphene dispersion (Figure 9).

**Table 1:** Developed Graphene-Based Formulations for Screen Printing Processing

graphene batch	binders	filler:binder	C_N-rGO_ (g/L)
N-rGO 1100	PVP	1:3	75
	PVDF	1:2	
	SEBS	2:3	
		1:1	

The different N-rGO material formulations were obtained by varying the binder itself (PVP, SEBS, and PVDF) using various filler:binder ratios (Table 1), while keeping the same concentration of graphene (75 g/L), corresponding to a filler content of about 6 wt % of the total ink weight.

The samples were printed with a homemade screen-printing setup with a metallic frame base structure at laboratory conditions (about 22 °C and 40% relative humidity). All the samples were printed using a 100T screen mesh with the screen placed at 1 mm of distance from the substrate: a commercial Kapton foil of 100 μm thickness and temperature resistance up to 400 °C from Archs company. The printed patterns were 1.5 × 70 mm pads and 20 × 20 mm squares. Five printing steps were used to evaluate the overall properties of the materials. After printing, the films were cured at 100 °C for 15 min in an oven (Binder E, model 28, Binder, Germany).

The thickness of the printed materials was measured using a mechanical profiler KLA Tencor D-100 (scan rate 0.05 mm/s, stylus force 0.2 mg, measured profile data averaged over a scan length of 600 μm). An average value of the thickness and roughness was calculated from three consecutive measurements.

### 4.3 Thermoforming Process

The mold conformation was carried out using a Vaquform DT2 desktop thermoformer. The mold consists of a 10 mm height × 20 mm base triagonal prism. First, the films were screen-printed using a 100T screen mesh onto 0.5 mm-thick polyethylene terephthalate glycol (PETG) sheets. Then, the conformation took place after reaching 150 °C for approximately 5 min, the temperature at which the polymer substrate started to deform, by pressing down onto the mold in vacuum mode for 30 s.

### 4.4 Characterization Techniques

The printed films were evaluated with a Leica EZ4 magnifier and by scanning electron microscopy (SEM) using a Hitachi S-4800 field emission SEM at an accelerating voltage of 5 kV with magnifications of 700× in cross-sectional mode. All samples were previously metalized with a 20 nm-thick gold layer deposited with a Polaron SC502 sputter coater.

Raman spectroscopy (inVia, Renishaw) was performed at an excitation wavelength of 514 nm in the range 150–3500 cm^–1^. X-ray photoelectron spectra (XPS) were carried out with a K-Alpha spectrometer (Kratos AXIS Supra) equipped with a monochromatic Al Kα source operated at 120 W. General survey spectrum scans and selected regions of interest were collected. Hybrid-slot lens mode was used, which corresponds to a spot analysis area of approximately 700 μm × 300 μm.

Indentation tests were carried out using a Micro Materials NanoTest with a Diamond indenter tip. The indentation tests were performed in a displacement control mode, in which the indenter tip displacement rate was 0.40 mN/s and the indentation depth was 20 mN.

The electrical resistance of the printed films was obtained by four-probe measurements using a Keithley 2400 source measurement unit. The samples were evaluated at three different points for each sample. The voltage was measured while applying an electrical current to the film, and the electrical resistivity (ρ) was calculated using eq 1:
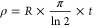
1where *R* is the film resistance, calculated by the inverse of the slope of the *I*(*V*) function, *t* is the thickness, calculated by profilometry, and  is the geometrical correction factor for the 20 × 20 mm squares and  for the pads.^52^ The four probes have a diameter of 0.9 mm, and the distance between them is 2 mm.

Piezoresistive measurements were carried out under mechanical bending (universal testing machine, Shimadzu AG-IS, load cell of 500 N) in *four-point-bending* mode.^53^ The electrical resistance of the samples was measured using an Agilent 34401A multimeter, while the mechanical bending was applied. The piezoresistive response was quantified by the gauge factor (GF) using eq 2:
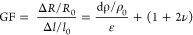
2where *R* is the electrical resistance, Δ*R* is the relative variation of the resistance, ρ is the electrical resistivity, ε = Δ*l*/*l*_0_, where *l* is the deformation, and ν is the Poisson ratio. In the four-point-bending mode, the deformation (ε) is given by eq 3,^19^
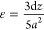
3where *z* is the vertical deformation, *d* the thickness of the sample, and *a* is the distance between the support bending points.

Indirect cytotoxicity evaluation of the samples was performed adapting the ISO 10993-5 standard test method. For that, L929 cells were cultured in 75 cm^2^ cell culture flask at 37 °C in a humidified environment and 5% CO_2_, using Dulbecco’s modified Eagle’s medium (DMEM, Biochrom, Berlin, Germany) containing 4.5 g.L^–1^ glucose, 10% fetal bovine serum (FBS, Biochrom, Berlin, Germany), and 1% (v/v) penicillin/streptomycin solution (P/S, Biochrom).

The different samples were cut with an area of 1 cm^2^ and sterilized by exposition of both sides of the samples to ultraviolet radiation for 1 h and washing with sterile phosphate-buffered saline solution (PBS, pH 7.4).

Then, a suspension of 3 × 10^4^ cell mL^–1^ was seeded in 96-well tissue culture polystyrene plates and incubated for 24 h at the same conditions described above, to ensure cell attachment on the plate. Simultaneously, each sample was incubated for 24 h in a 24-well tissue culture polystyrene plate. After this time, the cell culture medium was removed from the 96-well plates, and 100 μL of culture medium (previously in contact with the different samples) was added to each well and allowed to incubate for 72 h in standardized culture conditions, as mentioned above. A solution of 20% dimethyl sulfoxide (DMSO) was used for positive control and DMEM for negative control. The metabolic activity was evaluated after 72 h of incubation using the (3-(4,5-dimethylthiazol-2-yl)-5-(3-carboxymethoxyphenyl)-2-(4-sulfophenyl)-2*H*-tetrazolium) (MTS, Promega) assay. Briefly, the medium of every well was removed, and fresh medium containing MTS solution (in a 1:5 ratio) was added to each well and incubated for 2 h. After this incubation time, the optical density was measured at 490 nm with a spectrophotometric plate reader (Biotech Synergy HT).

The results are presented as the average of viability ± standard deviation. The percentage of cell viability was calculated according to eq 4.^54^
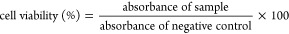
4
